# Supplementary material for: Measuring user interactions with websites: A comparison of two industry standard analytics approaches using data of 86 websites
Source: PLoS One. 2022 May 27;17(5):e0268212. doi: 10.1371/journal.pone.0268212 (PMC9140287; doi:10.1371/journal.pone.0268212)
Supplement: S1 File — (DOCX) [file pone.0268212.s001.docx]

# S1 Appendix 1 Supporting information: Website descriptions

S1 Table 1: Website Descriptions

| **Website** | **Site Type** | **Country** | **Website Category** |
| --- | --- | --- | --- |
| 1 | other | United States | Business_and_Consumer_Services/Business_Services |
| 2 | content | United States | Computers_Electronics_and_Technology/Computers_Electronics_and_Technology |
| 3 | other | United States | Computers_Electronics_and_Technology/Computers_Electronics_and_Technology |
| 4 | content | United States | Community_and_Society/Faith_and_Beliefs |
| 5 | content | Japan | News_and_Media |
| 6 | other | United Kingdom | Science_and_Education/Universities_and_Colleges |
| 7 | content | Belarus | News_and_Media |
| 8 | content | United States | News_and_Media |
| 9 | other | United States | Home_and_Garden |
| 10 | other | United States | News_and_Media |
| 11 | content | United States | Arts_and_Entertainment/Performing_Arts |
| 12 | content | United States | News_and_Media |
| 13 | content | Belgium | Vehicles/Vehicles |
| 14 | other | United States | Arts_and_Entertainment/TV_Movies_and_Streaming |
| 15 | other | United States | Health/Childrens_Health |
| 16 | other | Cuba | News_and_Media |
| 17 | other | United States | Computers_Electronics_and_Technology/Programming_and_Developer_Software |
| 18 | other | United States | Science_and_Education/Libraries_and_Museums |
| 19 | other | Taiwan | Computers_Electronics_and_Technology/Computers_Electronics_and_Technology |
| 20 | other | United States | News_and_Media |
| 21 | content | United States | Arts_and_Entertainment/Arts_and_Entertainment |
| 22 | content | Madrid, Spain | News_and_Media |
| 23 | other | United States | Jobs_and_Career/Jobs_and_Employment |
| 24 | content | India | Finance/Finance |
| 25 | transactional | United States | Computers_Electronics_and_Technology/Consumer_Electronics |
| 26 | other | United Kingdom | Gambling/Lottery |
| 27 | other | Spain | Computers_Electronics_and_Technology/Programming_and_Developer_Software |
| 28 | content | Israel | Finance/Finance |
| 29 | other | Ecuador | Gambling/Lottery |
| 30 | other | United States | Arts_and_Entertainment/Animation_and_Comics |
| 31 | content | Istanbul, Turkey | News_and_Media |
| 32 | content | Germany | Lifestyle/Fashion_and_Apparel |
| 33 | other | Japan | Vehicles/Makes_and_Models |
| 34 | content | United States | News_and_Media |
| 35 | other | United States | Computers_Electronics_and_Technology/Computer_Security |
| 36 | other | United States | Business_and_Consumer_Services/Marketing_and_Advertising |
| 37 | content | United States | Computers_Electronics_and_Technology/Computers_Electronics_and_Technology |
| 38 | other | United States | Science_and_Education/Biology |
| 39 | other | Noida, India | E-commerce_and_Shopping/Marketplace |
| 40 | content | United States | News_and_Media |
| 41 | content | United States | News_and_Media |
| 42 | content | Israel | News_and_Media |
| 43 | other | Japan | Hobbies_and_Leisure/Photography |
| 44 | content | Chile | News_and_Media |
| 45 | content | Malaysia | News_and_Media |
| 46 | other | United States | Arts_and_Entertainment/Humor |
| 47 | other | United States | Science_and_Education/Environmental_Science |
| 48 | other | France | Travel_and_Tourism/Travel_and_Tourism |
| 49 | other | United Kingdom | Science_and_Education/Universities_and_Colleges |
| 50 | other | United States | Travel_and_Tourism/Travel_and_Tourism |
| 51 | content | New Delhi, India | News_and_Media |
| 52 | content | Russian Federation | Arts_and_Entertainment/TV_Movies_and_Streaming |
| 53 | content | France | News_and_Media |
| 54 | content | United States | News_and_Media |
| 55 | content | United Kingdom | Community_and_Society/LGBTQ |
| 56 | content | Ukraine | News_and_Media |
| 57 | content | Japan | News_and_Media |
| 58 | content | Nigeria | News_and_Media |
| 59 | content | Russian Federation | News_and_Media |
| 60 | content | United States | Law_and_Government/Law_and_Government |
| 61 | other | United States | Community_and_Society/Philanthropy |
| 62 | content | United States | News_and_Media |
| 63 | other | United States | Computers_Electronics_and_Technology/Social_Networks_and_Online_Communities |
| 64 | content | Madagascar | News_and_Media |
| 65 | content | United States | Arts_and_Entertainment/Arts_and_Entertainment |
| 66 | content | India | Lifestyle/Beauty_and_Cosmetics |
| 67 | content | United States | Sports/Sports |
| 68 | content | United States | News_and_Media |
| 69 | other | United States | Finance/Finance |
| 70 | content | Russian Federation | News_and_Media |
| 71 | content | India | News_and_Media |
| 72 | content | Canada | News_and_Media |
| 73 | content | United States | Community_and_Society/LGBTQ |
| 74 | content | United States | News_and_Media |
| 75 | other | India | E-commerce_and_Shopping/Marketplace |
| 76 | content | Russian Federation | News_and_Media |
| 77 | content | United States | News_and_Media |
| 78 | content | United States | News_and_Media |
| 79 | content | Russian Federation | News_and_Media |
| 80 | content | France | News_and_Media |
| 81 | content | Israel | News_and_Media |
| 82 | transactional | China | Computers_Electronics_and_Technology/Programming_and_Developer_Software |
| 83 | other | United States | Arts_and_Entertainment/Books_and_Literature |
| 84 | content | United States | News_and_Media |
| 85 | content | Russian Federation | Arts_and_Entertainment/Humor |
| 86 | other | United Arab Emirates | Finance/Financial_Planning_and_Management |

# S2 Appendix 2 Supporting information: Website analytics Google Analytics

S2 Table 1: Website Analytics Google Analytics

| **Website** | **Average  Monthly Visits** | **Average  Monthly Unique Visitors** | **Average  Monthly Bounce Rate** | **Average Session Duration Monthly** |
| --- | --- | --- | --- | --- |
| 1 | 3152687.583 | 2461417.75 | 0.470219118 | 943.9167 |
| 2 | 8110386.667 | 6186346.833 | 0.775873246 | 754.5000 |
| 3 | 14311548.5 | 8821303 | 0.661145647 | 228.4167 |
| 4 | 23426176.67 | 14554697.42 | 0.82525285 | 99.2500 |
| 5 | 27033752.25 | 20306072.08 | 0.736031486 | 975.5000 |
| 6 | 850428.4167 | 509126.75 | 0.421168727 | 121.4167 |
| 7 | 5214572.917 | 2923813.75 | 0.240343213 | 708.0000 |
| 8 | 10692195.33 | 7656292.417 | 0.178776099 | 221.0833 |
| 9 | 2779309.75 | 2286648.083 | 0.612926326 | 116.1667 |
| 10 | 802915.5 | 757859.75 | 0.765019882 | 407.5000 |
| 11 | 4929489.083 | 3883782.833 | 0.730989242 | 591.8333 |
| 12 | 48905992.17 | 39635508 | 0.592168472 | 129.8333 |
| 13 | 21523123.08 | 15293159.75 | 0.72934511 | 177.5000 |
| 14 | 2176824.667 | 1309846 | 0.233196396 | 80.8333 |
| 15 | 1595396.083 | 1421402.917 | 0.733490719 | 815.6667 |
| 16 | 6921366.833 | 3249242.167 | 0.002929683 | 388.2500 |
| 17 | 2576940.833 | 3888423.583 | 0.580858548 | 204.3333 |
| 18 | 1100232.167 | 894130.6667 | 0.545703848 | 648.5833 |
| 19 | 1628533.917 | 1071823 | 0.284254189 | 893.0000 |
| 20 | 12757798.25 | 6383750.917 | 0.757184692 | 99.7500 |
| 21 | 27179097.08 | 20323785.42 | 0.547136358 | 305.0833 |
| 22 | 19166469.42 | 12122846.58 | 0.795311054 | 586.0000 |
| 23 | 91652.83333 | 79775 | 0.865318648 | 327.5000 |
| 24 | 28482891 | 19707050.92 | 0.805026506 | 3292.3333 |
| 25 | 870787.0833 | 710426.25 | 0.335160224 | 353.2500 |
| 26 | 51557365.08 | 5687884.917 | 0.388996176 | 970.8333 |
| 27 | 34541387.92 | 16014184.08 | 0.498814671 | 270.0000 |
| 28 | 10564186.75 | 3429957 | 0.537760825 | 226.0833 |
| 29 | 1983614.833 | 982909.8333 | 0.558179529 | 397.7500 |
| 30 | 7456951.917 | 1857211.583 | 0.329622639 | 1617.9167 |
| 31 | 111602489.5 | 46731059 | 0.529078261 | 280.0000 |
| 32 | 6813004.667 | 5135493.25 | 0.555012906 | 420.1667 |
| 33 | 12182444.92 | 6910290.083 | 0.463394074 | 364.6667 |
| 34 | 6352498.25 | 2207956.5 | 0.486415967 | 109.0833 |
| 35 | 1997.666667 | 1799.083333 | 0.664537042 | 286.3333 |
| 36 | 229965 | 166798.9167 | 0.607758405 | 236.3333 |
| 37 | 243532.9167 | 206969.6667 | 0.889157866 | 170.3333 |
| 38 | 1343039.417 | 666781.75 | 0.471746615 | 157.0000 |
| 39 | 51678665.67 | 31594844.33 | 0.315127463 | 62.4167 |
| 40 | 88510097.17 | 52104710.83 | 0.7256284 | 880.0000 |
| 41 | 24366953.25 | 11936003.42 | 0.762132212 | 282.1667 |
| 42 | 13965468.67 | 7871127.833 | 0.58131945 | 166.2500 |
| 43 | 559129.5833 | 466001.3333 | 0.607726982 | 273.3333 |
| 44 | 47680925.83 | 19526373.17 | 0.752953197 | 821.0000 |
| 45 | 12645211.42 | 4312074 | 0.726669815 | 237.0833 |
| 46 | 2759598.083 | 2067727.25 | 0.865426319 | 283.7500 |
| 47 | 3907998.167 | 3159884.25 | 0.74980537 | 190.7500 |
| 48 | 2139992.75 | 1629079.167 | 0.677615623 | 110.7500 |
| 49 | 759081.8333 | 409214.25 | 0.400115462 | 1893.6667 |
| 50 | 6186609.25 | 3361710.25 | 0.341500258 | 651.3333 |
| 51 | 292590329.6 | 138161008.9 | 0.570733706 | 594.2500 |
| 52 | 24500224.58 | 12513015.83 | 0.678529272 | 478.3333 |
| 53 | 18068651.58 | 10453201.92 | 0.70983938 | 159.7500 |
| 54 | 59412725.08 | 27218653.75 | 0.718552377 | 217.2500 |
| 55 | 5699329.917 | 4173119.5 | 0.822845583 | 407.5000 |
| 56 | 33693450.58 | 8629271.5 | 0.607701456 | 86.6667 |
| 57 | 24812334.58 | 19576788.5 | 0.87537632 | 157.8333 |
| 58 | 18018003.67 | 5277602 | 0.607224413 | 291.4167 |
| 59 | 6731533.083 | 4062760.833 | 0.829681269 | 65.3333 |
| 60 | 11293072.75 | 4352797.583 | 0.5926922 | 206.1667 |
| 61 | 2281485.583 | 1380645.083 | 0.509298981 | 305.4167 |
| 62 | 7094639.5 | 5812068.917 | 0.622874487 | 239.0000 |
| 63 | 196389 | 177928.9167 | 0.296991586 | 368.2500 |
| 64 | 24521584.92 | 9364105.417 | 0.455343191 | 120.5833 |
| 65 | 573115.25 | 189210.6667 | 0.532903218 | 2807.5833 |
| 66 | 17498653.67 | 11986710.17 | 0.205373871 | 319.3333 |
| 67 | 12304290.5 | 6589191.25 | 0.720107654 | 367.5833 |
| 68 | 15516234.5 | 4462750.167 | 0.530748342 | 102.3333 |
| 69 | 11426337 | 9149060.167 | 0.406976657 | 176.9167 |
| 70 | 6758686.5 | 4300593.667 | 0.692686137 | 1433.0833 |
| 71 | 7492234.917 | 4516944 | 0.764277465 | 559.8333 |
| 72 | 2051345.25 | 1102865.917 | 0.709920914 | 256.0000 |
| 73 | 1804926.25 | 990478.6667 | 0.026372066 | 87.9167 |
| 74 | 16218226.25 | 5906764.417 | 0.629027739 | 96.8333 |
| 75 | 4357188.833 | 2984500.25 | 0.180870127 | 255.4167 |
| 76 | 6211631.167 | 3152871.667 | 0.651405679 | 119.0833 |
| 77 | 1751255.667 | 1006191.667 | 0.201107244 | 137.9167 |
| 78 | 99996425.5 | 38793027.17 | 0.24228531 | 243.5000 |
| 79 | 19717282.08 | 12815426.58 | 0.65954501 | 264.2500 |
| 80 | 5493372.167 | 3643043.417 | 0.574981188 | 335.3333 |
| 81 | 87167442.33 | 8420241.417 | 0.332171805 | 161.0000 |
| 82 | 39059975.5 | 28558115.5 | 0.716272917 | 173.9167 |
| 83 | 14598658 | 1829401.5 | 0.332638747 | 199.9167 |
| 84 | 15516234.5 | 4462750.167 | 0.530748342 | 540.4167 |
| 85 | 11695221.42 | 2874024.083 | 0.373375388 | 125.5833 |
| 86 | 1148937.167 | 892821.1667 | 0.71231198 | 727.7500 |

# S3 Appendix 3 Supporting information: Website analytics SimilarWeb

S3 Table 1: Website Analytics SimilarWeb

| **Website** | **Average  Monthly Visits** | **Average  Monthly Unique Visitors** | **Average  Monthly Bounce Rate** | **Average Session Duration Monthly** |
| --- | --- | --- | --- | --- |
| 1 | 2388907.765 | 1723510.478 | 0.522239943 | 943.9167 |
| 2 | 6370707.286 | 4461316.249 | 0.733540587 | 228.4167 |
| 3 | 5721655.141 | 3382191.178 | 0.687457971 | 99.2500 |
| 4 | 12120851.27 | 6796965.02 | 0.703572252 | 975.5000 |
| 5 | 15203014.22 | 10656861.69 | 0.722715623 | 121.4167 |
| 6 | 935449.2818 | 317374.3113 | 0.380212185 | 708.0000 |
| 7 | 3681880.702 | 1842679.887 | 0.670640928 | 116.1667 |
| 8 | 5068416.047 | 4064305.28 | 0.859660416 | 407.5000 |
| 9 | 2692270.947 | 2082194.822 | 0.603059848 | 591.8333 |
| 10 | 765050.7068 | 345349.0386 | 0.622945633 | 129.8333 |
| 11 | 2865494.018 | 2030999.32 | 0.737941698 | 177.5000 |
| 12 | 34579903.79 | 26737357.77 | 0.796744957 | 80.8333 |
| 13 | 14811676.87 | 8973264.019 | 0.626186849 | 815.6667 |
| 14 | 2164885.174 | 1159374.679 | 0.54817656 | 388.2500 |
| 15 | 761146.4321 | 579862.9406 | 0.802073979 | 204.3333 |
| 16 | 2867211.391 | 1195611.79 | 0.572779414 | 648.5833 |
| 17 | 7010920.766 | 2709755.091 | 0.40366145 | 893.0000 |
| 18 | 716159.7037 | 560744.3371 | 0.790737415 | 99.7500 |
| 19 | 3183954.772 | 1956432.115 | 0.505406662 | 305.0833 |
| 20 | 8228777.224 | 3872027.51 | 0.671093644 | 586.0000 |
| 21 | 20274415.4 | 13179973.22 | 0.790241534 | 327.5000 |
| 22 | 10491888.5 | 6419772.311 | 0.653590866 | 3292.3333 |
| 23 | 103009.1337 | 68855.27846 | 0.821516349 | 353.2500 |
| 24 | 15798598.98 | 9593024.534 | 0.727064685 | 970.8333 |
| 25 | 900465.0495 | 596305.1576 | 0.61630397 | 270.0000 |
| 26 | 58302016.85 | 2886727.894 | 0.695345646 | 226.0833 |
| 27 | 6885892.37 | 2583460.509 | 0.51443352 | 397.7500 |
| 28 | 8232854.198 | 1767752.007 | 0.567546422 | 1617.9167 |
| 29 | 1322541.842 | 712351.6277 | 0.583107092 | 280.0000 |
| 30 | 9352918.241 | 1110239.692 | 0.288115669 | 420.1667 |
| 31 | 74736928.02 | 24097820.92 | 0.485934029 | 364.6667 |
| 32 | 4054221.627 | 2898342.365 | 0.769083179 | 109.0833 |
| 33 | 8981312.127 | 4478757.908 | 0.470243263 | 286.3333 |
| 34 | 4108930.231 | 695383.9647 | 0.48769502 | 236.3333 |
| 35 | 4442.509111 | 2361.047803 | 0.517420751 | 170.3333 |
| 36 | 265155.6379 | 154976.3899 | 0.575520701 | 157.0000 |
| 37 | 210800.0379 | 142743.539 | 0.838767545 | 62.4167 |
| 38 | 1362674.899 | 509150.6034 | 0.375376749 | 880.0000 |
| 39 | 37371608.22 | 22157759.3 | 0.403739888 | 282.1667 |
| 40 | 39072175.27 | 21528677.94 | 0.677407205 | 166.2500 |
| 41 | 7593530.087 | 4992986.955 | 0.691076353 | 273.3333 |
| 42 | 9635567.227 | 4202373.22 | 0.582539838 | 821.0000 |
| 43 | 724034.1739 | 268164.7597 | 0.38921257 | 237.0833 |
| 44 | 22623953.74 | 8257865.119 | 0.664172232 | 283.7500 |
| 45 | 6065159.63 | 2079656.493 | 0.67944628 | 190.7500 |
| 46 | 1671898.758 | 1224237.27 | 0.851664178 | 110.7500 |
| 47 | 2183370.285 | 1613172.693 | 0.828404567 | 1893.6667 |
| 48 | 1257324.605 | 854909.3785 | 0.654379033 | 651.3333 |
| 49 | 2204373.081 | 571216.3292 | 0.401262583 | 594.2500 |
| 50 | 4446304.631 | 2012046.222 | 0.391229252 | 478.3333 |
| 51 | 140842047.4 | 54603245.75 | 0.575970445 | 159.7500 |
| 52 | 20769969.86 | 11289247.85 | 0.672062517 | 217.2500 |
| 53 | 10117014.26 | 5099486.616 | 0.711560857 | 407.5000 |
| 54 | 35210421.76 | 15529483.4 | 0.681587948 | 86.6667 |
| 55 | 3104726.451 | 2278108.797 | 0.776031658 | 157.8333 |
| 56 | 27525245.18 | 5501082.757 | 0.586115616 | 291.4167 |
| 57 | 15243146.61 | 10507876.26 | 0.792547863 | 65.3333 |
| 58 | 19257988.95 | 4557307.938 | 0.567932423 | 206.1667 |
| 59 | 4515748.479 | 2727169.607 | 0.747331958 | 305.4167 |
| 60 | 6483665.102 | 1466650.748 | 0.512183966 | 239.0000 |
| 61 | 2064108.573 | 1111328.176 | 0.652972561 | 368.2500 |
| 62 | 2663199.815 | 2212343.244 | 0.842867014 | 120.5833 |
| 63 | 53682.1421 | 25878.40882 | 0.509211765 | 367.5833 |
| 64 | 9177501.383 | 4437700.434 | 0.748692196 | 102.3333 |
| 65 | 36462.28147 | 17950.97715 | 0.621652334 | 176.9167 |
| 66 | 8503973.014 | 6113216.932 | 0.69941467 | 1433.0833 |
| 67 | 8116216.82 | 3357472.596 | 0.627499369 | 559.8333 |
| 68 | 19669962.33 | 2168608.631 | 0.532891004 | 256.0000 |
| 69 | 7572406.521 | 5642262.017 | 0.775997243 | 87.9167 |
| 70 | 4691076.992 | 3093788.549 | 0.799526726 | 96.8333 |
| 71 | 5204326.055 | 2993246.828 | 0.653054931 | 255.4167 |
| 72 | 1172428.124 | 611089.3792 | 0.746872759 | 119.0833 |
| 73 | 1222692.484 | 523555.5913 | 0.620594311 | 137.9167 |
| 74 | 12045793.11 | 2466255.438 | 0.513376909 | 243.5000 |
| 75 | 2566371.935 | 1686171.771 | 0.641341493 | 264.2500 |
| 76 | 5485904.187 | 2375686.82 | 0.692431541 | 161.0000 |
| 77 | 812444.345 | 435586.5934 | 0.753511888 | 173.9167 |
| 78 | 45030665.15 | 15328262.17 | 0.584333186 | 199.9167 |
| 79 | 12703039.59 | 7572706.231 | 0.664091991 | 540.4167 |
| 80 | 2233017.103 | 1515350.543 | 0.771036806 | 125.5833 |
| 81 | 35705163.5 | 3665306.285 | 0.473086111 | 727.7500 |
| 82 | 28943766.98 | 22458142.35 | 0.720372669 | 140.8333 |
| 83 | 17038080.16 | 1083361.775 | 0.350634155 | 4498.0833 |
| 84 | 19669962.33 | 2168608.631 | 0.532891004 | 256.0000 |
| 85 | 31608794.38 | 4829207.487 | 0.309169263 | 997.0000 |
| 86 | 699477.0247 | 454446.5339 | 0.751216377 | 390.4167 |
